# Skin microbiota dynamics following *B. subtilis* formulation challenge: an *in vivo* study in mice

**DOI:** 10.1186/s12866-021-02295-y

**Published:** 2021-08-21

**Authors:** Veronica Moskovicz, Rina Ben-El, Guy Horev, Boaz Mizrahi

**Affiliations:** 1 Faculty of Biotechnology and Food Engineering, Technion - Israel Institute of Technology, 3200003 Haifa, Israel; 2 Bioinformatics Knowledge Unit, The Lorry I. Lokey Interdisciplinary Center for Life Sciences and Engineering, Technion - Israel Institute of Technology, 3200003 Haifa, Israel; 3 Faculty of Biology, Technion - Israel Institute of Technology, 3200003 Haifa, Israel

**Keywords:** *Bacillus subtilis*, Live bacterial delivery, Pluronic F-127, Microbiota, Skin

## Abstract

**Background:**

Modulating the microbiota is a leading-edge strategy for the restoration and maintenance of a healthy, balanced environment. The use of health-promoting bacteria has demonstrated some potential benefits as an alternative for skin microbiota intervention. Here, we investigate the manipulation of mice skin microbiota using B. subtilis incorporated into a supportive Pluronic F-127 hydrogel formulation. The formula plays an important role in delivering the bacteria to the desired action site.

**Results:**

The *B. subtilis* challenge induced a shift in the composition and abundance of the skin microbiota. Containment of *B. subtilis* in the Pluronic F-127 hydrogel accelerated bacterial modulation compared with free *B. subtilis*. The abundance of both *Staphylococcus* and *Corynebacterium spp.* was altered as a result of the live bacterial intervention: the abundance of *Corynebacterium* increased while that of *Staphylococcus* decreased. Four days after last application of the *B. subtilis* formulation, *B. subtilis* counts returned to its initial level.

**Conclusions:**

B. subtilis intervention can induce a shift in the skin microbiota, influencing the abundance of commensal, beneficial, and pathogenic bacteria. Containment of *B. subtilis* in Pluronic hydrogel accelerates the microbial alteration, probably by facilitating bacterial attachment and supporting continuous growth. Our results reveal the ability of *B. subtilis* in Pluronic to modulate the skin microbiota composition, suggesting that the formulation holds therapeutic potential for skin disease treatment.

**Supplementary Information:**

The online version contains supplementary material available at 10.1186/s12866-021-02295-y.

## Background

The skin, our largest organ, serves as the principal mechanical and biological protective barrier for the human body. Being the organ most exposed to the environment, the skin is colonized by a diverse collection of microorganisms that constitute the microbiota [1]. By communicating with epithelial cells and the immune system, the microbiota plays an important role in protecting the skin from potential damages posed by pathogenic microorganisms [2, 3]. Moreover, findings of the Human Microbiome Project have revealed that many of the resident microorganisms are harmless (commensals) or have a positive influence on human skin health and well-being (mutualists) [4, 5]. However, when homeostasis is disrupted, the microbiota enters into a state of microbial imbalance, termed dysbiosis, which can lead to dermal immune dysregulation [6, 7]. Skin microbiota dysbiosis has been associated with skin diseases including atopic dermatitis [8], acne [9], and vitiligo [10].

Several strategies for the manipulation of the skin microbiota have been suggested, including hygiene products [11], antibiotics [12, 13], and prebiotics [14]. Extrinsic factors, ranging from cosmetics to the environment and antibacterial agents, as forces that impact the human skin microbiome and well-being, were recently reviewed [15]. These methods, however, often suffer from poor efficacy and lack of selectivity towards the pathogenic bacteria [16]. Antibiotics, for example, have been associated with long-term bacterial imbalances, lasting up to several years and leading to an increased incidence of skin lesions [17, 18]. Antibiotic treatment has been showed to enhance depigmentation in vitiligo-affected skin [13] and to delay wound healing [19]. These drawbacks are driving the exploration of new microbiota intervention alternatives.

The use of health-promoting bacteria has shown promising results in the restoration of a healthy microbiota, for example by promoting the growth of beneficial microbes [20–23]. *Bacillus subtilis* has potential as a microbiota-modulating agent since it can efficiently outcompete important human pathogens such as E. coli and S. aureus [24, 25], probably through the production and secretion of potent antimicrobial agents [25] while displaying a non-pathogenic profile [24, 26, 27]. Moreover, B. subtilis efficiently outcompetes important human pathogens such as E. coli and S. aureus in vitro [24, 25], probably through the production and secretion of potent antimicrobial agents [25]. However, before this knowledge can be translated into therapeutic applications, additional groundwork is required and well-controlled *in vivo* studies to assess microbiota dynamics as well as safety and efficacy aspects of living bacteria interventions must be performed. The aim of this study is, therefore, to explore the experimental manipulation of the skin microbiota using a *B. subtilis* formulation incorporated into a supportive Pluronic F-127 hydrogel delivery matrix. Pluronic F-127 Poly (ethylene oxide)-poly(propylene oxide)-poly(ethylene oxide)) was selected as the main matrix due to its lower critical solution temperature, around body temperature, since it allows bacillus to grow inside the formula and prolonged the retention of B. subtilis on the skin without compromising bacteria’s ability to produce and secret its wide range of potent antimicrobial agents [28]. The microbiota of healthy skin was mapped before, during, and after the administration of a living *B. subtilis* formulation to monitor its dynamics. We hypothesized that a *B. subtilis* microbiota intervention will result in a microbial shift that will be limited to the treatment course, since human skin microbiota is relatively stable in terms of its microbial population [29, 30]. One possible explanation for skin microbiome stability may well be that transient bacteria do not tend to establish themselves permanently on the skin, but rather persist only for hours to days [31].

## Results

**Fig. 1:**
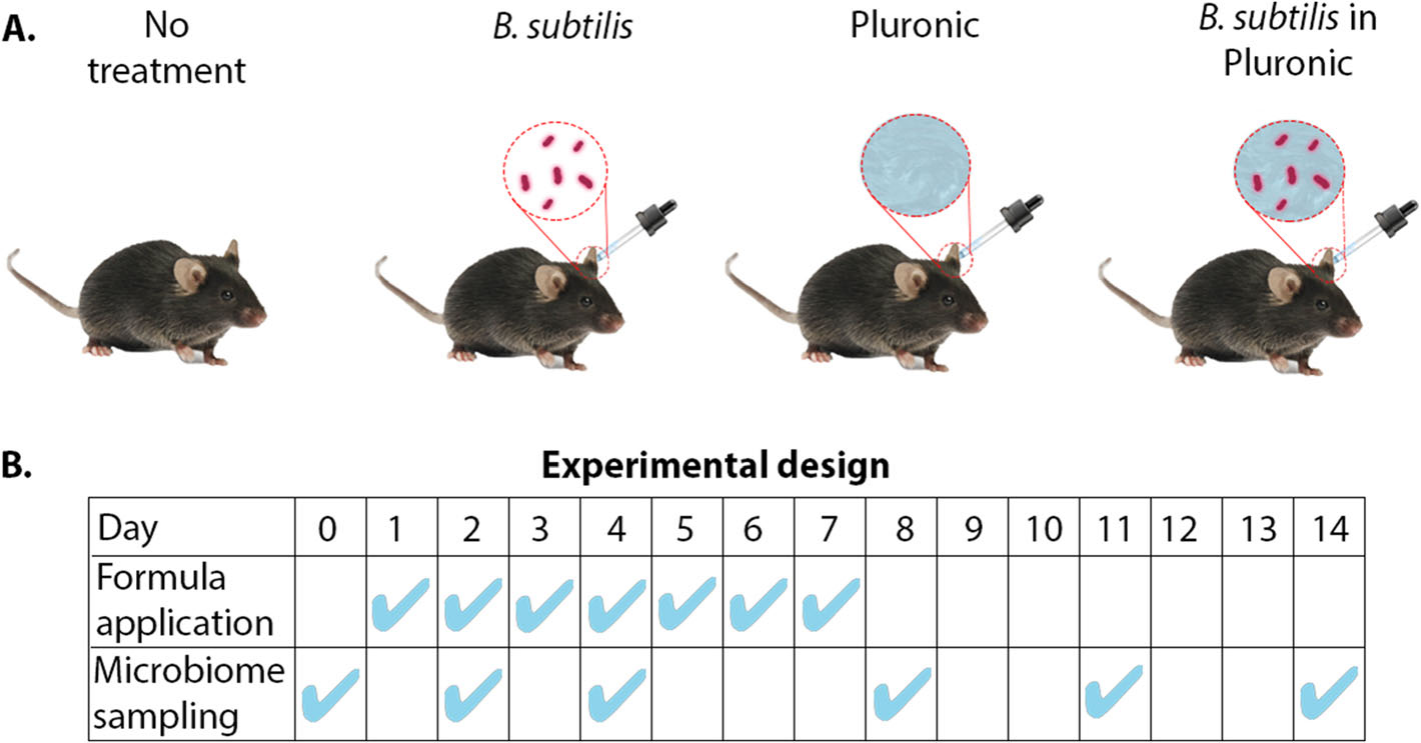
Experimental design. **A **Four groups of six C57BL/6 female mice each received different treatments: no treatment (control), *B. subtilis*, plain Pluronic hydrogel, and *B. subtilis* in Pluronic hydrogel. **B** Formulas were applied twice daily for seven days. Skin samples were collected for microbiota analysis on days 0, 2, 4, 8, 11, and 14

The effects of challenging the healthy mouse ear skin microbiota with a B. subtilis formulation were studied using the ear skin of laboratory mice. This procedure has been used as a model system for human skin sites in terms of morphology and microbiota and has been successful in assessing host-microbe interactions [32, 33]. Twenty-four 8-week old C57BL/6 female mice were randomly assigned to one of four groups: Pluronic hydrogel containing *B. subtillis*, *B. subtillis* (in LB medium), plain Pluronic hydrogel, and a no-treatment control group (Fig. 1A). Each group was administered with the corresponding formulation twice a day for seven days. The effects of the various treatments were analyzed by determining the bacterial composition of the skin before the first application (day 0) and on days 2, 4, 8, 11, and 14 (Fig. 1B). Of note, all animals did not show any sign of discomfort or changes in general behavior.

After skin sampling, genomic DNA was extracted and the V3-V4 hypervariable regions of 16 S rRNA gene were amplified and sequenced using the Illumina technology. A total of 29.5 million high-quality 16 S rRNA gene sequences were obtained, each containing between 0.36 and 1.5 % of the data. Noise was removed according to the Remove Unwanted Variation (RUV) strategy [34], using the untreated control group and day 0 samples (before treatment) for normalization (Supplementary Table 1).

**Fig. 2:**
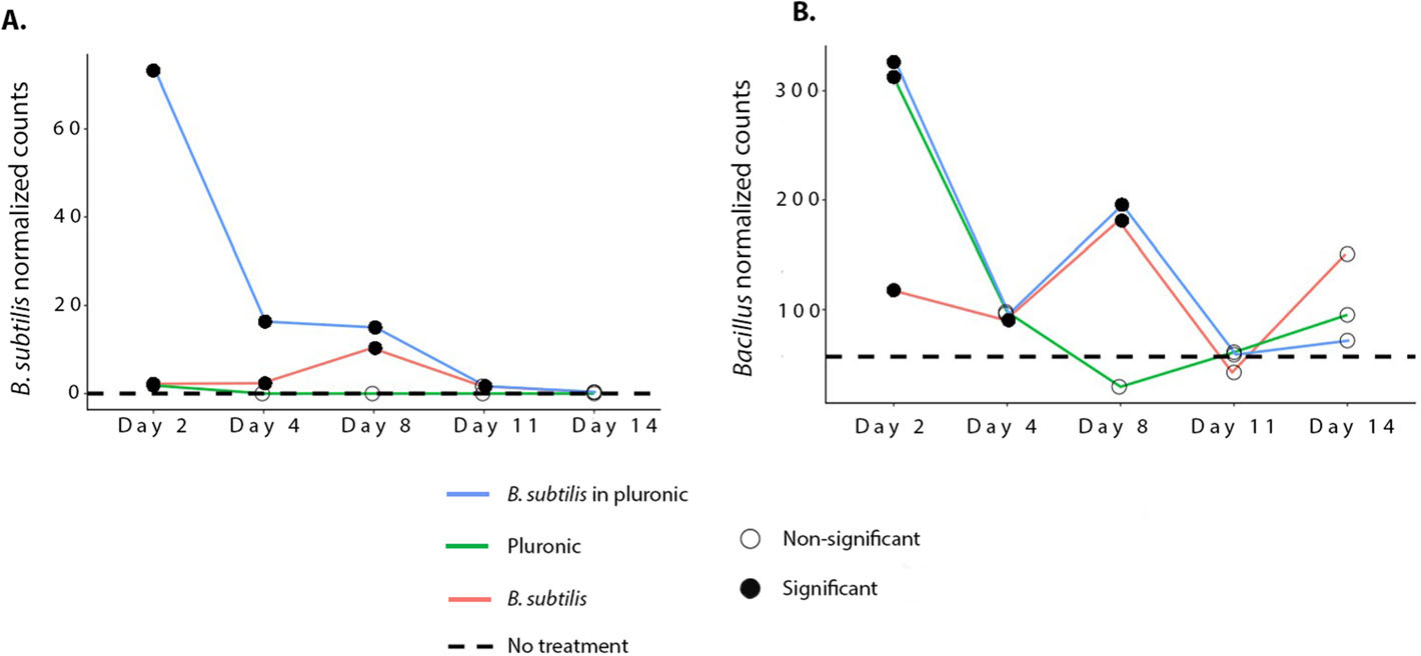
Temporal and treatment-dependent alteration of *Bacillus subtilis* species (**A**) and *Bacillus* genus (**B**). Dashed line represents average counts of control samples. *Bacillus* and *B. subtilis* counts that differ statistically significantly from control (padj < 0.05, Wald-test) are denoted by solid circles, while empty circles represent lack of significant difference

The dynamics of the abundance of *B. subtilis*, in particular, and of the *Bacillus* genus, in general, before, during, and after administration of *B. subtilis* formulations was assessed (Fig. 2A and B) and compared with the average abundances of *B. subtilis* and *Bacillus* (dashed lines), respectively. Indeed, plain Pluronic hydrogel did not impact the abundance of *B. subtilis*, as no B. subtilis was present in untreated samples (Fig. 2A). *B. subtilis* in Pluronic, conversely, had the highest influence on *B. subtilis* counts on days 2 and 4, which were significantly higher than for the *B. subtilis* treatment (Log_2_ differential expression 6.6 and 3.6 respectively, padj < 0.05). Nevertheless, four days after the last administration, all groups presented counts that were similar to that of the untreated group. For the *Bacillus* genus (Fig. 2B), counts were significantly higher for all groups compared with the untreated control group: The group that received *B. subtilis* showed a 2-fold increase while the two groups that received Pluronic (with and without *B. subtilis*) exhibited a 6-fold increase. This trend changed on day 4, when all groups presented a two-fold increase compared with the untreated group. Post-challenge, on day 8, *Bacillus* counts for the two groups that received *B. subtilis* increased 4-fold, while counts for the group that received plain Pluronic decreased to the control group level. From day 11, i.e. 4 days after the last administration, control levels were attained for all treatment groups with insignificant differences compared with the untreated group.

**Fig. 3:**
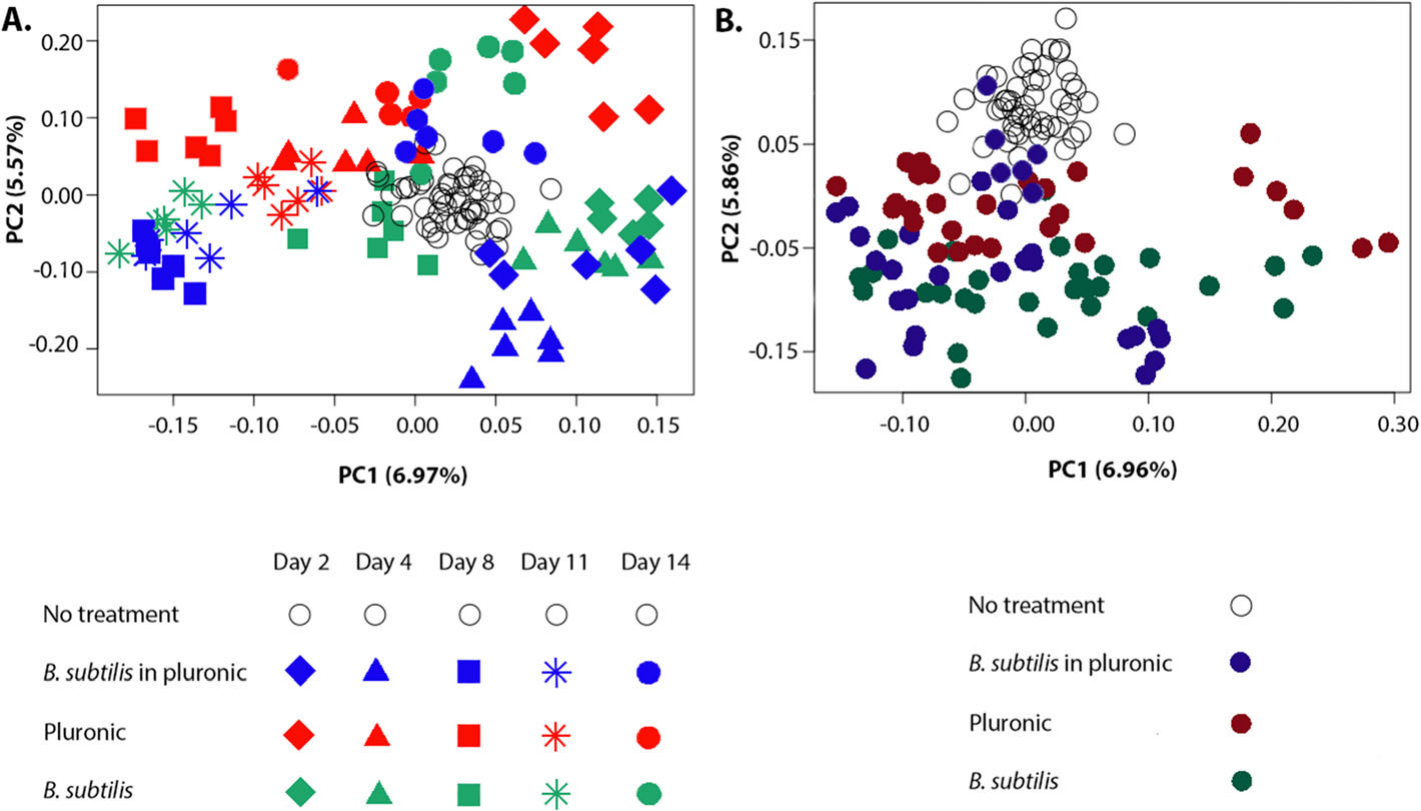
Principal components analysis (PCA) of skin bacterial communities at the genus (**A**) and species (**B**) levels. Axes explain the effect of timing (PC1) and treatment (PC2) on the observed changes

PCA was then used to assess skin microbiota dynamics on both the genus (Fig. 3A) and the species (Fig. 3B and Supplementary Fig. 1) levels. Our data indicated a clear clustering according to treatment day and nature as explained by PC1 and PC2, respectively (i.e., data organization according to treatment nature along PC2 axis can be observed in Fig. 3B). Treatment nature and time point influenced the observed clustering to the same extent, as evidenced by the similarity in effect size between PC1 and PC2 (6.97-6.96 % and 5.57-5.86 %, respectively). The *B. subtilis* in Pluronic group exhibited an enhanced microbial shift compared with the pure *B. subtilis* group, which showed a very similar pattern but with a slight delay, prominent on day 8 (Fig. 3A). One week after ceasing treatment administration, however, all groups presented a microbiota composition similar to that of the untreated control group.

**Fig. 4:**
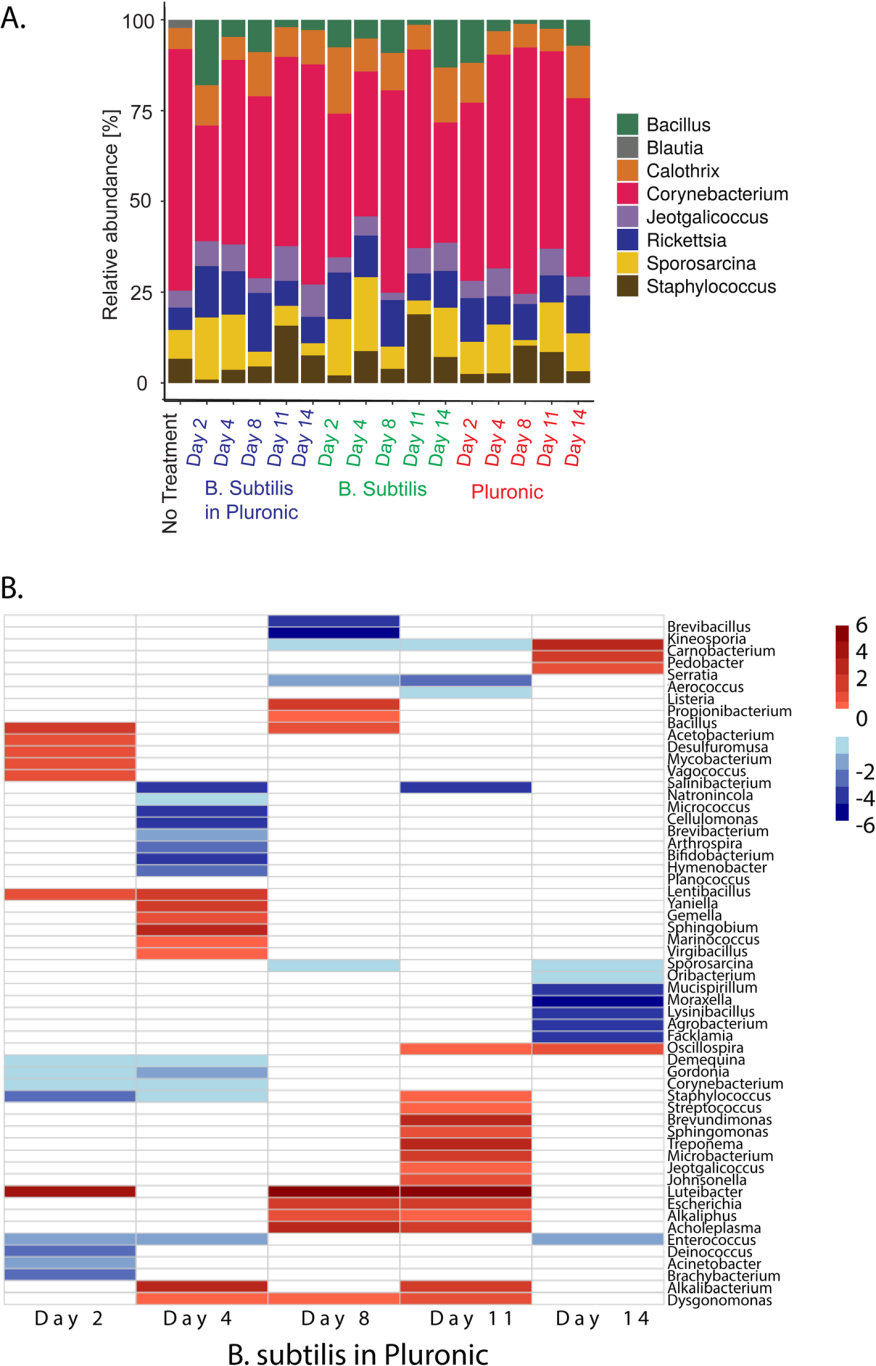
Effect of *B. subtilis* challenge on the microbial composition of the skin. **A** Relative abundance plot for the most represented bacterial genera in the ear skin microbiota. **B** Differential representation of bacterial genera along treatment with *B. subtilis* in Pluronic hydrogel compared with the untreated control group (**B**). Statistically significant differences in bacterial abundance were considered (-1 < log2FC > 1 ;padj < 0.05, Wald-test)

We further investigated the microbiota shift upon *B. subtilis* intervention and after its cessation by analyzing the intervention’s effects on the relative abundance of the most represented skin bacterial genera (Fig. 4A and Supplementary Table 2). Consistent with the PCA results, altered bacterial abundance was observed for all treatment groups along the experiment. For *Corynebacterium*, the most abundant genera in the ear skin microbiota, application of *B. subtilis* in Pluronic resulted in a sharp increase from day 2 to 4, followed by a plateau for the remainder of the experiment. Application of pure *B. subtilis* caused an abundance increase only on day 8, which remained until day 11 and then decreased to initial values. No significant variations in abundance were observed following the application of pure Pluronic. Interestingly, the relative abundance of *Staphylococcus* exhibited an inverse trend compared with *Bacillus* for both *B. subtilis*-containing formulations: when *Bacillus* abundance increased, *Staphylococcus* counts decreased, and vice versa (Spearman rank correlation rho=-0.6769414, *p* = 4.5*10^− 13^). To obtain a broader view of these bacterial changes, we mapped the differential representation of bacterial genera along the treatment period (Fig. 4B). Only statistically significant differences in bacterial abundance (-1 < log2FC > 1; padj < 0.05, Wald-test) were considered for the analysis (Supplementary Table 3). Following the administration of *B. subtilis* in Pluronic, several highly related genera were observed to cluster, being either underrepresented or overrepresented compared with the untreated control group (Fig. 4B). For instance, the *Lentibacillus*, *Gemella*, *Marinococcus*, and *Virgibacillus* genera, of the *Bacilliales* order, were overrepresented on day 4. The *Bacillus* genus, on the other hand, was overrepresented on days 2 and 8, while no statistically significant difference to the control was presented on day 4, consistent with the trend observed in the PCA (Fig. 2B). *Staphylococcus* abundance decreased during the application of *B. subtilis* in Pluronic formulation (days 2 and 4) and increased on day 11, three days after the last administration.

## Discussion

The concept of skin microbiota manipulation, either by promoting bacterial balance or by pathogen inhibition, is well established [35, 36]. However, despite significant progress in the field, transplantation of bacteria or bacterial ingredients that selectively stimulate or inhibit the growth and activity of one or a limited number of bacterial species is still at a very experimental stage in skin therapy [4]. Local delivery of live bacterial therapies is often challenging. Live bacterial therapy is often challenging as a local delivery system since the bacteria must reach the site of action alive and establish themselves there [15], hence the importance of proper formulation design. Moreover, the effect of the live-bacteria formulation observed here was limited to treatment course. Skin microorganisms play an essential role in maintaining many aspects of human health including protection against pathogens, education of our immune system, and the breakdown of natural products [2]. Since these aspects of skin microbiota are not fully understood, a transient, temporary effect may be viewed as an advantageous over a stable, long-term modulation.

In this study, administration of *B. subtilis* to the ear skin of mice resulted in the modulation of the skin microbial composition. Our results indicate that a suitable dermal delivery system is of prime importance for successful administration of live bacteria and, consequently, for microbiota modulation. Administration of *B. subtilis* in Pluronic hydrogel resulted in a significant increase in *B. subtilis* counts compared with a more moderate increase in bacterial levels in the absence of Pluronic. Significant differences (p < 0.05) was found between *B. subtilis* in Pluronic and *B. subtilis* groups on days 2 and 4 but not on day 8. The ability of B. subtilis to support the growth of other members of the genus Bacillus was explained by cell-cell interactions that induce biofilm production and by the immunity of bacillus to molecules secreted from closely related bacteria [37]. The effect of Pluronic hydrogel on the *Bacillus* genera was significant during the first two days of application: the two Pluronic groups (pure Pluronic and *B. subtilis* in Pluronic) showed a fast increase in *Bacillus* abundance that surpassed the effect exhibited by the pure *B. subtilis* group. The enhanced performance of the B. subtilis in Pluronic formulation can be explained by the potential contribution of various factors. One is the ability of Pluronic F-127 to selectively reduce the attachment and biofilm formation of several bacteria [38], probably owing to its surfactant properties [39]. Another explanation is that Pluronic F-127 gel serves as a protective layer between the bacteria and the skin, aiding the delivery of B. subtilis to the desired site. Pluronic F-127 was found to be very effective in reducing the degradation of such peptides while sustaining their delivery from the hydrogel to its surroundings [40, 41]. Finally, the ability of Pluronic gel to enhance the immune response, probably by stimulating the expression of vascular epithelial growth factors, may be related to the selective shift in the skin microbial composition [42]. We note that the differences in microbiome composition can also be attributed to specific displacement of certain microorganisms or to an overall decrease in microbiota abundance. Nevertheless, the present study is, to our knowledge, among the first to evaluate the use of biomaterials in live bacterial skin delivery systems for microbiota modulation. The application of *B. subtilis* altered the abundance of common skin bacteria, including *Staphylococcus* and *Corynebacterium* spp. Some of these bacterial genera have been reported to be the most abundant organisms colonizing moist areas of the skin, including the antecubital fossa in humans, which the ear skin of mice resembles [43, 44]. Common skin commensals belonging to the *Corynebacterium* genus have been shown to exhibit antimicrobial activity against human pathogens and to stimulate healthy host-bacterial interactions [45]. For example, *C. striatum* can influence *S. aureus* gene expression by downregulating virulence-related genes and upregulating genes associated with the establishment of a commensal relationship [46].

## Conclusions

In this study, we investigated the effect of challenging the skin microbiota with *B. subtilis*, a bacterium with therapeutic potential [24, 25, 27]. The carrier, a Pluronic F-127 hydrogel, was found to facilitate *B. subtilis* administration and enhance its activity, possibly by supporting continuous bacterial growth and providing better conditions for skin attachment. The interaction between the polymer, *B. subtilis*, and epithelial cells should be further investigated to provide more insight into the mechanism of action. We demonstrated that *B. subtilis* induces a shift in the skin microbiota composition that is facilitated by the presence of the live bacteria in Pluronic hydrogel. This alteration was characterized by a shift in the skin microbiota, influencing the abundance of commensal, beneficial and pathogenic bacteria. Given the great potential of the live bacterial delivery approach, its clinical value for the treatment of conditions associated with skin microbiota dysbiosis (i.e. atopic dermatitis or acne) should be investigated.

## Methods

### Animal husbandry

Twenty-four 8-week old C57BL/6 female mice were purchased from Envigo, Israel. We restricted the experiment to female mice in order to avoid any possible interference of sex factors with the variability of microbial flora. The animals were caged randomly in four groups of six mice each. All animals were maintained in sterilized cages on a 12-h light/12‐h dark cycle with food and water provided ad libitum. Bedding was changed once a week, and mice were given an autoclaved chow diet and sterilized water. Moderate dermal irritation or 20 % body weight loss were used to determine the humane endpoints. At the end of the study, mice were sacrificed by CO_2_ asphyxiation following protocols approved by the corresponding authority.

### Formulations and administration

Each independently housed group of six mice received a different treatment formulation twice daily, every 12 h, for 7 days. B. subtilis 3610 was chosen since it is a natural, non-modified wild type strain. The treatment formulations consisted of (a) 10 % v/v *B. subtillis* in lysogeny broth (LB) with 18 % w/v Pluronic (“*B. subtilis* formulation”), (b) 18 % w/v Pluronic, (c) 10 % v/v *B. subtillis* in LB, and (d) untreated control. A stock of Pluronic F-127 was prepared by dissolving the appropriate amount of polymer in distilled water to obtain a final concentration of 20 % w/v. A stock solution of *B. subtilis* was cultured in LB agar and incubated at 37 °C overnight after which a bacterial colony was transferred to a falcon tube containing fresh liquid LB, incubated at 37 °C and allowed to reach an optical density (OD) of 0.6 at 600 nm. The *B. subtilis* formulation was prepared by adding 0.5 mL of fresh bacterial culture (OD_600_: 0.6) to 4.5 mL of 20 % w/v Pluronic, obtaining a final solution of 18 % w/v Pluronic with 10 % v/v *B. subtilis* in LB. 18 % w/v Pluronic was obtained by adding 0.5 mL of liquid LB to 4.5 mL of Pluronic 20 % w/v. To prepare 10 % v/v *B. subtillis* in LB, 0.5 mL of fresh bacterial culture (OD_600_: 0.6) were added to 4.5 mL of fresh LB media. The different formulations (100 µL) were administered to both left and right ears of the animals of the corresponding groups. Pluronic-containing formulations were allowed to harden on the skin for 1 min until a viscous gel was obtained.

### Sample collection

To sample the skin microbiota, both ears were thoroughly swabbed with a sterile FLOQSwab presoaked in buffer solution (0.15 M NaCl and 0.1 % Tween 20). Sampling was effectuated every other day from day 0, before formulation administration, to day 14, a week after last application. Samples were stored at -80 °C until processing.

### Bacterial DNA extraction

DNA extraction and purification were performed using the PureLink Microbiome Kit (Invitrogen, Thermo Fisher Scientific) according to manufacturer’s protocol and supplementary instructions for low bio-burden samples. Concentration of purified DNA was determined using the QuBit High Sensitivity DNA quantification system (Invitrogen) and stored at 20 °C until further use.

### 16 S rRNA gene amplification and sequencing

16 S rRNA gene amplification and sequencing were carried out at the Technion’s Genome Center. Sequencing libraries were prepared using the 16 S rRNA Metagenomic Sequencing Library Preparation protocol by Illumina with minor adjustments. Sample input was 0.625 ng of genomic DNA and the first PCR amplification consisted of 30 cycles. V3 and V4 hypervariable regions of bacterial 16 S rRNA were the amplification targets. Primers used to target the V3 and V4 regions of the 16 S rRNA gene were 5’TCGTCGGCAGCGTCAGATGTGTATAAGAGACAGCCTACGGGNGGCWGCAG (Forward) and 5’GTCTCGTGGGCTCGGAGATGTGTATAAGAGACAGGACTACHVGGGTATCTAATCC (Reverse). The respective Illumina overhang adapter sequences were included in the primer design. All 96 libraries were sequenced on an Illumina MiSeq instrument with 250 paired-ends reads. Sequencing data was input into the 16 S rRNA gene Metagenomics app on the Illumina BaseSpace sequence hub. Classification was performed using the Illumina 16 S rRNA gene Metagenomics workflow, which includes demultiplexing of indexed reads, FASTQ files generation, and read classification. Operational Taxonomic Unit (OTU) clustering and classification were performed at genus and species level. An Illumina-curated version of the Greengenes database was used as the taxonomy database for the metagenomics workflow. The algorithm used is a high-performance implementation of the Ribosomal Database Project Classifier described in Wang Q. et al. [47].

### Bioinformatics analyses

The R statistical software package was used for all statistical tests. The Remove Unwanted Variation (RUV) normalization strategy described by Risso et al. [34] was employed to remove noise from unknown sources. Factors of unwanted variation using replicate samples were estimated using the RUVseq package. Parameters were set to K = 15 at the species level and K = 40 at the genus level, and corrected counts were calculated. For the calculations, the no-treatment control treatment was defined as all no-treatment samples and all day-0 samples (before treatment). Principal components analysis (PCA) was applied on RUVseq corrected counts with Euclidean distance as similarity metric using EDAseq [48]. A design matrix including both the covariates of interest and the factors of unwanted variation was supplied to DESeq2 for differential analysis [49]. To test the effect of treatments, each treatment was compared with the no treatment control using Wald test and the False Discovery Rate correction for multiple comparisons as implemented in DESeq2 [49]. Only bacteria with at least 50 counts in 2 or more samples were included in the analysis. A bacterial genus or species was considered to differ significantly between treatments if the absolute value fold change was at least 2, with an adjusted *p*-value (padj) smaller than 0.05.

## Supplementary Information

**Additional file 1: Supplementary Figure 1.** Principal coordinates analysis (PCA) of skin bacterial communities at species level. Each treatment group and treatment day is distinguished by a different color. Empty dots represent control samples.

**Additional file 2.**

**Additional file 3: Supplementary Table 2.** Relative abundance of the seven most represented bacterial genera for different treatments (B. subtilis in Pluronic, B. subtilis and Pluronic) and treatment days (2, 4, 8, 11 and 14). Each experimental group consisted of 6 mice, named A-F for replicates. For each bacterial genus, given a treatment and treatment day, the mean and relative abundance in percentage (Rel. Ab. (%)) are calculated.

**Additional file 4: Supplementary Table 3.** Differential bacterial species resulting from *B. subtilis* in Pluronic treatment on different treatment time points, identified by DESeq2 (-1<log2FC>1 ;padj<0.05, Wald-test). Only bacteria with at least 50 counts in two or more samples were included in the analysis. 

## Data Availability

The datasets generated and/or analysed during the current study are available in the BioProject database repository (BioProject ID PRJNA640303): https://www.ncbi.nlm.nih.gov/bioproject/640303. Original R scripts are available in GitHub under request.

## Abbreviations

LB: Lysogeny broth
OD: Optical density
OTU: Operational taxonomic unit
PCA: Principal component analysis
PCR: Polymerase chain reaction
RUV: Remove unwanted variation
