# Supplementary material for: Skin microbiota dynamics following B. subtilis formulation challenge: an in vivo study in mice
Source: BMC Microbiol. 2021 Aug 21;21:231. doi: 10.1186/s12866-021-02295-y (PMC8379746; doi:10.1186/s12866-021-02295-y)

**Supplementary Figure 1**: Principal coordinates analysis (PCA) of skin bacterial communities at species level. Each treatment group and treatment day is distinguished by a different color. Empty dots represent control samples.


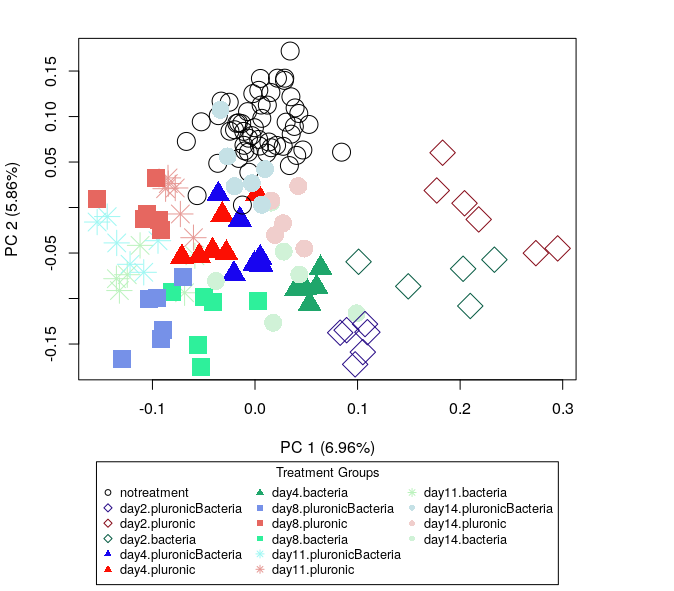

Supplement: Supplementary file 1 — Additional file 1: Supplementary Figure 1. Principal coordinates analysis (PCA) of skin bacterial communities at species level. Each treatment group and treatment day is distinguished by a different color. Empty dots represent control samples. [file 12866_2021_2295_MOESM1_ESM.docx]
